# Autogenous Demineralized Dentin Graft With High Molecular Weight Hyaluronic Acid in Ridge Preservation: Pilot Trial

**DOI:** 10.1111/cid.70100

**Published:** 2025-11-14

**Authors:** Rahma Hesham, Nesma Shemais, Heba Ahmed Saleh, Karim Fawzy El‐Sayed

**Affiliations:** ^1^ Oral Medicine and Periodontology Department, Faculty of Dentistry Cairo University Cairo Egypt; ^2^ Oral and Maxillofacial Pathology Department, Faculty of Dentistry Cairo University Cairo Egypt; ^3^ Clinic for Conservative Dentistry and Periodontology, School of Dental Medicine, Christian Albrechts University Kiel Germany; ^4^ Stem Cells and Tissue Engineering Research Unit, Faculty of Dentistry Cairo University Cairo Egypt

**Keywords:** autograft, clinical trial, dentin, extraction, hyaluronic acid, tooth

## Abstract

**Objective:**

The current trial assessed for the first time radiographic and histological alterations, following alveolar ridge preservation (ARP), using autogenous demineralized dentin graft carried in 0.2% high molecular weight sodium hyaluronate (ADDG+HA; test group) versus autogenous demineralized dentin graft (ADDG, control group) alone.

**Material and Methods:**

Thirty patients (*n* = 30) with non‐restorable single‐rooted teeth were randomly assigned into two groups (*n* = 15/group). Following extraction, ARP was performed using either ADDG solely or ADDG+HA. Bucco‐lingual alveolar ridge width (BLRW; primary outcome), buccal (BRH) and lingual ridge height (LRH), percentage of newly formed bone, soft tissue and residual graft in human biopsies histologically, as well as patients' pain and discomfort (all secondary outcomes) were assessed after 6 months at the time of implant placement. Sample bone core biopsies were further collected, processed, and histomorphometrically and SEM analyzed.

**Results:**

For the ADDG and ADDG+HA groups, the alveolar ridge dimensional changes were comparable, being −1.21 ± 0.77 mm and −1.18 ± 0.86 mm in BLRW, −0.89 ± 0.74 and −0.83 ± 0.85 mm in BRH, and −0.9 ± 0.76 mm and −1.05 ± 1.18 mm in LRH respectively (*p* > 0.05). Clinically, no complications, pain, or inflammatory responses were reported. Histologically, all samples demonstrated bone growth and socket bone fill, while the ADDG+HA group showed a significantly greater presence of mineralized mature bone, which accounted for 33% ± 8.1% of the specimen after 6 months.

**Conclusions:**

Both ADDG and ADDG+HA demonstrated comparable outcomes in terms of ARP. HA amalgamation with ADDG appears to enhance bone mineralization and maturation, yet without a significant impact on dimensional changes during ARP procedures.

**Trial Registration:** NCT05613075

## 1 Introduction

Following tooth extraction, a chain of biological events is initiated, reducing the alveolar process's dimensions and causing variable degrees of alveolar ridge resorption [1, 2]. These dimensional changes are especially evident during the initial 3 months following extraction, remain ongoing for years [3], and could complicate the functional and esthetic outcomes of a subsequent prosthetic rehabilitation [4]. Thus, in the endeavors for the preservation of the alveolar ridge (ARP) in postextraction sockets, to hinder (even if partially) such bone loss [5], a wide range of grafting materials, including autografts, xenografts, alloplasts and allografts were suggested [6].

Despite possessing osteogenic and osteo‐inductive potentials [7], autogenous bone suffers from a high rate of resorption, with a possibility of significant donor site morbidity, in addition to restricted intraoral availability [8]. In this context, extracted teeth, which are typically discarded as biological waste, were suggested as an autogenous graft alternative, relying on the biological resemblance of dentin and alveolar bone matrices [9, 10, 11]. According to radiographic diffraction analysis, the inorganic component of dentin, which accounts for 70% of the dentin's weight volume, is composed of low‐crystalline calcium phosphate, comparable to the alveolar bone [12], which osteoclasts can break down for efficient bone remodeling [13, 14], a feature crucial for ARP.

Hyaluronic acid (HA), a fundamental component of the extracellular matrix (ECM) is one of nature's most hygroscopic compounds. When HA is introduced to an aqueous solution, adjacent carboxyl and N‐acetyl groups establish hydrogen bonds, allowing HA to retain water and preserve conformational rigidity [15]. Through mesenchymal cell chemotaxis, proliferation and subsequent differentiation, HA accelerates bone formation and repair [16, 17]. It further elevates osteogenesis and osteoblastic differentiation [18]. High molecular weight (HMW)‐HA also produces a hydrogel‐like matrix that stabilizes clots and facilitates cell migration, making it a viable tool for the process of bone regeneration [19]. In addition to its advantages in bone healing, HA further offers anti‐inflammatory properties and promotes angiogenesis [20, 21]. In the field of ARP, HA was suggested to foster dentoalveolar regeneration [22], and especially when combined with deproteinized bovine bone mineral to provide significant benefits in reducing bone resorption and preserving bone width [21]. Thus, it remains plausible to assume that a combination of HA with ADDG could provide similar results for ARP.

To the best of the authors' knowledge, limited clinical evidence currently exists in the literature on the adjunctive effects of HA on dentin grafts in ARP procedures. Hence, the current pilot randomized controlled trial's objective was to assess the radiographic bucco‐lingual alveolar ridge width (primary outcome), buccal and lingual ridge heights, along with postoperative pain and patient satisfaction, as well as the histological quality of newly regenerated bone and SEM analysis (all secondary outcomes) when using autogenous demineralized dentin graft (ADDG) with HA versus ADDG solely for ARP.

## 2 Materials and Methods

### 2.1 Study Design and Registration

The current pilot randomized clinical trial was designed as a uni‐center, double‐blinded, active‐controlled, parallel‐group study with a 1:1 allocation ratio in accordance with EQUATOR criteria. The Research Ethics Committee of Cairo University's Faculty of Dentistry, Egypt authorized the research protocol, informed consent forms, and requests for biological sample collection (IRB approval number:13|11|22), in November 2022. The study's protocol was recorded on www.clinicaltrials.gov in November 2022 (NCT05613075). The study was carried out in compliance with the ethical standards of the Helsinki Declaration for medical research involving human subjects as revised in Seoul, 2008.

### 2.2 Recruitment of Participants

The present study was conducted at the postgraduate Implantology and Periodontology Clinics, Faculty of Dentistry, Cairo University, Egypt. Motivated adults over the age of 18 years were recruited from the outpatient clinic, with a single‐rooted nonrestorable tooth/root due to fracture, unfavorable crown–root ratio, or extensive caries in the esthetic zone, with bleeding on probing percentage less than 15%, and plaque index of less than 15% [11, 23]. Thin labial bone plate of (≤ 1 mm) was ensured via CBCT prior to the start of the surgery. Following extraction, a UNC‐15 periodontal probe was used to check the socket walls and ensure the presence of type 1 subdivision B sockets inside the bony housing, with intact four walls [2, 24]. Smokers, patients with a history of chemotherapy, radiotherapy, or bisphosphonate therapy, patients with systemic conditions that could impair healing or bone metabolism, such as uncontrolled diabetes or hyperthyroidism, as well as pregnant women, and teeth with acute infection at the extraction site were excluded. All patients with a previous history of bone grafting at the surgical site were further eliminated.

### 2.3 Sample Size

To reject the null hypothesis, a sample size of 24 sites was required to detect an assumed relevant anticipated difference between groups of 0.5 mm in the bucco‐lingual alveolar ridge width (primary outcome), with 80% power, a 5% level of significance, and a standard deviation (SD) of 0.41 mm [25]. This number was raised to 30 sites (one site per patient) to compensate for a possible 20% dropout. Calculations were performed using PS Power and Sample Size Calculations software, version 3.1.2.

### 2.4 Randomization and Blinding

Using the online sequence generator (www.random.org) simple randomization was performed through generating numbers from 1:30 by an investigator (KF) who was not involved in recruitment. The allocation sequence was concealed from the operator (RA) and was revealed following tooth extraction, using sequentially numbered and sealed opaque envelopes. Extraction sites of the eligible participants who agreed to be included in the study were randomly assigned into two equal parallel groups (*n* = 15/group) with a 1:1 allocation ratio to receive ARP using either ADDG+HA (test group) or ADDG alone (control group) based on the generated sequence. Due to differences in techniques the operator (RA) could not be blinded to the procedure. Meanwhile, the participants, outcomes assessors (NS and HS) and the statistician were blinded.

### 2.5 Preoperative Phase

All patients who were recruited underwent full mouth professional mechanical plaque removal (PMPR). Oral hygiene instructions were thoroughly explained, along with directions to rinse twice a day for 2 weeks with 0.12% chlorohexidine HCl Mouthwash (Hexitol, The Arab Drug Company for Pharmaceutical and Chem. Ind. Co. Cairo, Egypt) for 2 weeks. Cone‐beam computed tomography (CBCT, Planmeca, Helsinki, Finland) was used to conduct radiographic analysis on the assigned arch, and all radiographic measures were recorded. The initial CBCT scan was carried out on the day of surgery and it was compared to a final CBCT that was scheduled to be performed 6 months postoperative, prior to the dental implant placement.

### 2.6 Surgical Procedures

Extractions in both groups were executed under local anesthesia, 2% mepivacaine HCl combined with 1:20 000 levonordefrin (Alexandria Co. for Pharmaceuticals, Alexandria, Egypt). Using surgical forceps and periotomes (Nordent Manufacturing Inc., IL, USA), minimally invasive atraumatic tooth/root extraction was carried out. Care was taken to prevent injury to the marginal bone, when forceps were further required. Using a UNC‐15 periodontal probe (Nordent Manufacturing Inc., IL, USA), the integrity of the extraction socket walls was examined.

#### 2.6.1 Control‐Group

**FIGURE 1:**
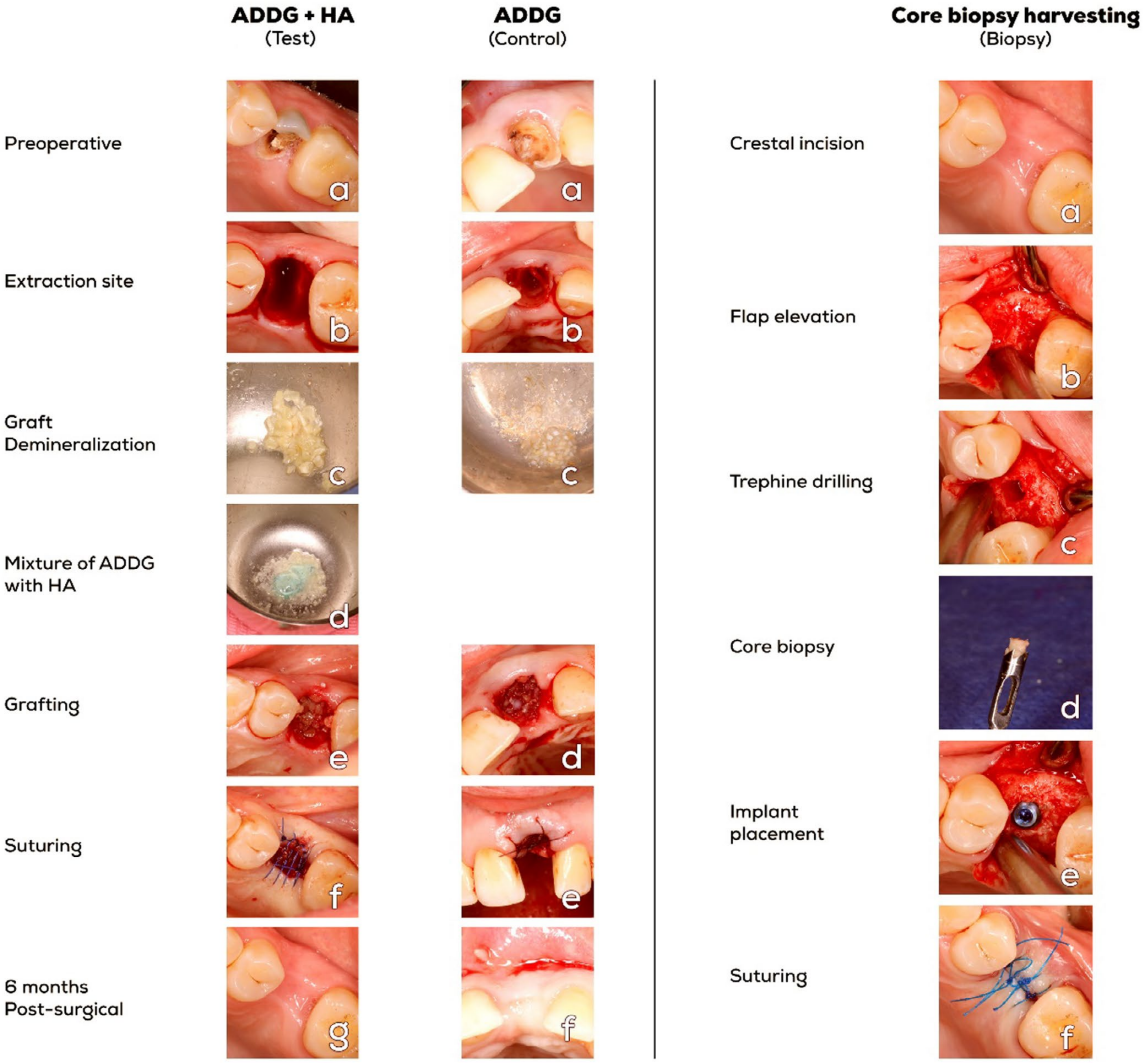
Clinical graft preparation, and grafting procedures at the ADDG in HA (test) (*left*), and the ADDG (control) (*middle*): (a) preoperative clinical photographs, (b) extraction sites, (c) tooth graft grinding and demineralization, (d) mixture of ADDG + HA, (e/d) tooth graft condensation into the socket, (f/e) suturing and wound securing. (g/f2) 6 months postoperative follow‐up. Graft Harvesting and Biopsy extraction and preservation (*right*): (a) Preoperative photograph, (b) flap incision and elevation, (c) trephining and harvesting form implant site, (d) Biopsy harvesting, (e) implant insertion, (f) suturing and wound closure.

Saline irrigation and a high‐speed fine finishing stone were used to remove periodontal ligaments from the extracted teeth, cementum, soft tissue attachment, any filling materials (gutta‐percha, composite, etc.), granulation tissues, or caries (if present). Sterile endodontic files and dental stones were also used to clean the pulp chamber. Extracted teeth were then ground into small pieces using a bone rongeur, and then a manual bone mill (Gold Bone Mill, MCT Bio, Seoul, Korea) was used to get a consistent micro‐sized range of tooth particles, as small as possible before the mill was not able to make them any smaller clinically. The resulting autogenous tooth particles were then demineralized in 0.6 N hydrochloric acid (Chemajet Chemicals, 6th of October, Egypt) for 30 min [11]. This was followed by two saline washes and drying with sterile gauze (Figure 1).

#### 2.6.2 Test‐Group

Following tooth extraction and preparation as explained in the control group, 2 mL Gengigel 0.2% HA (Ricerfarma, Via Egadi, Milano, Italy) was added and mixed with the ADDG graft material as a carrier.

In both groups, the graft was placed inside the socket, following irrigation of the extraction sockets with sterile saline. Using 4/0 mono‐filament polypropylene sutures (Demophorius Healthcare, Limassol, Cyprus), an internal crisscrossed knot was used to attach the bio‐absorbable collagen membrane (Hypro‐Sorb, Bioimplon, Gießen, Germany) over the grafted sockets (Figure 1).

### 2.7 Postoperative Care and Follow Up

Patients in both groups received instructions to prevent stress and brushing at the surgical site, to avoid interfering with the suture and to refrain from hot meals and forceful washing for a period of 24 h [11, 26]. For 2 weeks, a mouthwash with 0.12% chlorhexidine twice a day was used [27, 28] along with brushing of the remaining dentition. After 2 weeks from the day of surgery, brushing was advised for the full mouth, including the grafted zone. Following surgery, 500 mg of amoxicillin (Pharmaceuticals and Chemical Industries, Cairo, Egypt) was administrated three times daily for 10 days [29, 30], or 100 mg of doxycycline (Doxymycin, Nile Co. Alexandria, Egypt) twice a day for those who were sensitive to penicillin [29], along with 600 mg of ibuprofen (Brufen, Kahira Pharmaceuticals, Cairo, Egypt) for those who experienced strong pain [31]. Two weeks following the procedure, the sutures were removed. All postoperative treatment, patient satisfaction and pain scores were documented.

### 2.8 Outcomes

#### 2.8.1 Radiographic Outcomes

**FIGURE 2:**
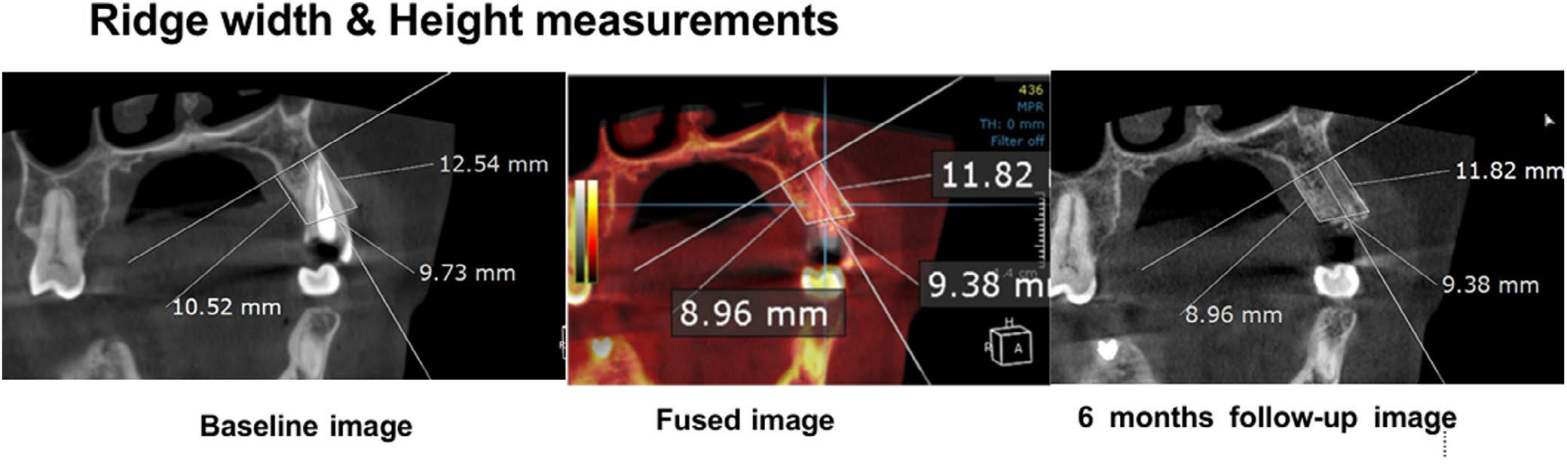
Radiographic examination, a CBCT at baseline (left) and 6 months postoperatively (right) with the fused radiographic cross‐section cut (middle).

An independent blinded calibrated examiner (NS) evaluated alterations in buccolingual ridge width (BLRW, primary outcome), buccal ridge height (BRH) and lingual ridge height (LRH, secondary outcomes) on CBCT images taken at baseline and 6 months after surgery. All scans were acquired using 0.4 mm voxel size, 90 kV and 8 mA. DICOM‐formatted scans were exported using a 3D viewer program (Blue Sky Plan 4.7.2, Blue Sky Bio, Illinois, USA). As previously described [10], at the mesio‐distal center of the extraction sites, matched slices of the baseline and final scans were obtained. In both cuts, the same dental reference points were placed (Ondemand 3D, Cybermed, 1.0.9, Seoul, South Korea) for standardized measurements. A bisecting vertical reference line that matched the long axis of the alveolar ridge was placed in the center of the extraction socket, while horizontal reference lines that were perpendicular to the extraction site were placed at its most apical point (Figure 2). BLRW was measured 2 mm below the most coronal cross section and perpendicular to the vertical reference line from the most coronal crest points to the horizontal reference line. Final data were subtracted from baseline values to reflect the changes in alveolar ridge dimensions, which were then represented in millimeters and percentages.

#### 2.8.2 Clinical Outcomes

##### 2.8.2.1 Postoperative Patient Satisfaction

Patient satisfaction was assessed on the same day of extraction after the completion of the surgical procedures, by asking the patients whether they were satisfied with the treatment provided, if they would do it again and whether they would recommend the treatment to others [32].

##### 2.8.2.2 Pain Scores

Pain was assessed at the suture removal appointment [33], where the patients reported any pain along the previous two weeks after the completion of surgical procedures using a 0–10 visual analogue scale (VAS) in which 0 means no pain while 10 represents the worst pain possible [31].

#### 2.8.3 Histological Analysis

**FIGURE 3:**
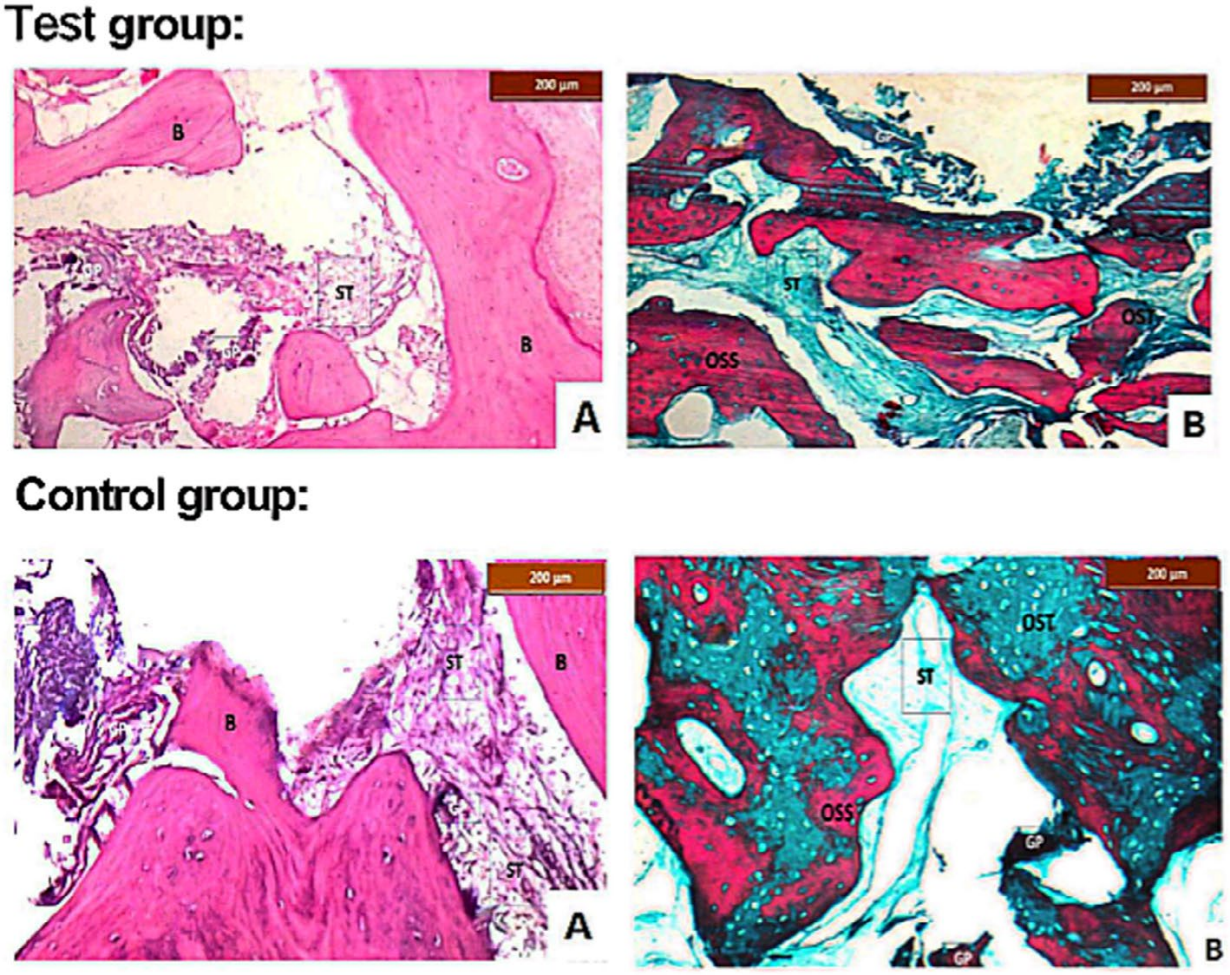
Qualitative histological analysis. Typical microscopic pictures (100× magnification) of histological sections of ADDG+HA (test, left) and ADDG (control, left) biopsies stained with hematoxylin and eosin. Masson trichrome staining of histological sections of ADDG+HA (test, right) and ADDG (control, right) biopsies. B: bone, GP: graft particles, OSS: osseous hard bone, OST: osteoid soft bone; ST: soft tissue.

Six months after engraftment, 14 bone samples were taken, seven from each group. Apart from the fact that some patients refused to place an implant or placed a fixed partial denture at the site at the time of samples' collection by the end of the follow‐up period, the histological analysis, similar to previous investigations [34, 35], did not include all patients as it was intended for demonstrative reasons, yet with an adequate number to allow for a possible qualitative/quantitative comparison. Human biopsies were taken using a trephine bur (Helmut Zepf Medizintechnik, Seitingen‐Oberflacht, Germany) from the center of the ARP site, with an outside diameter of 3.5 mm, an inner diameter of 3.0 mm, and a depth of 8–10 mm during implant insertion in grafted locations (Figure 3). Following processing in EDTA, samples were embedded in tissue blocks made of paraffin after being initially preserved in a 10% formalin solution. For staining purposes with either Masson's trichromatic (MT) or hematoxylin and eosin (H&E) for histological assessment and histomorphometry analysis, sections of the paraffin blocks, each 4 μm thick, were cut lengthwise. A light microscope (Leica Digital Microscope, Leica Microsystems, Wetzlar, Germany) was used to take photo‐micrographs for each sample. Image analyzer software (Leica QWin 500 image analysis program, Leica Microsystems, Balgach, Switzerland) was used to histomorphometrically estimate the percentage of the entire examined histological area that was made up of soft tissue stroma, graft particles and bone. The histological accessor (HS) was blinded to the samples and their respective groups. Using 100× magnification, the area % of newly produced bone was elected and measured in each sample in three consecutive fields.

### 2.9 Scanning Electron Microscopy (SEM) Analysis

**FIGURE 4:**
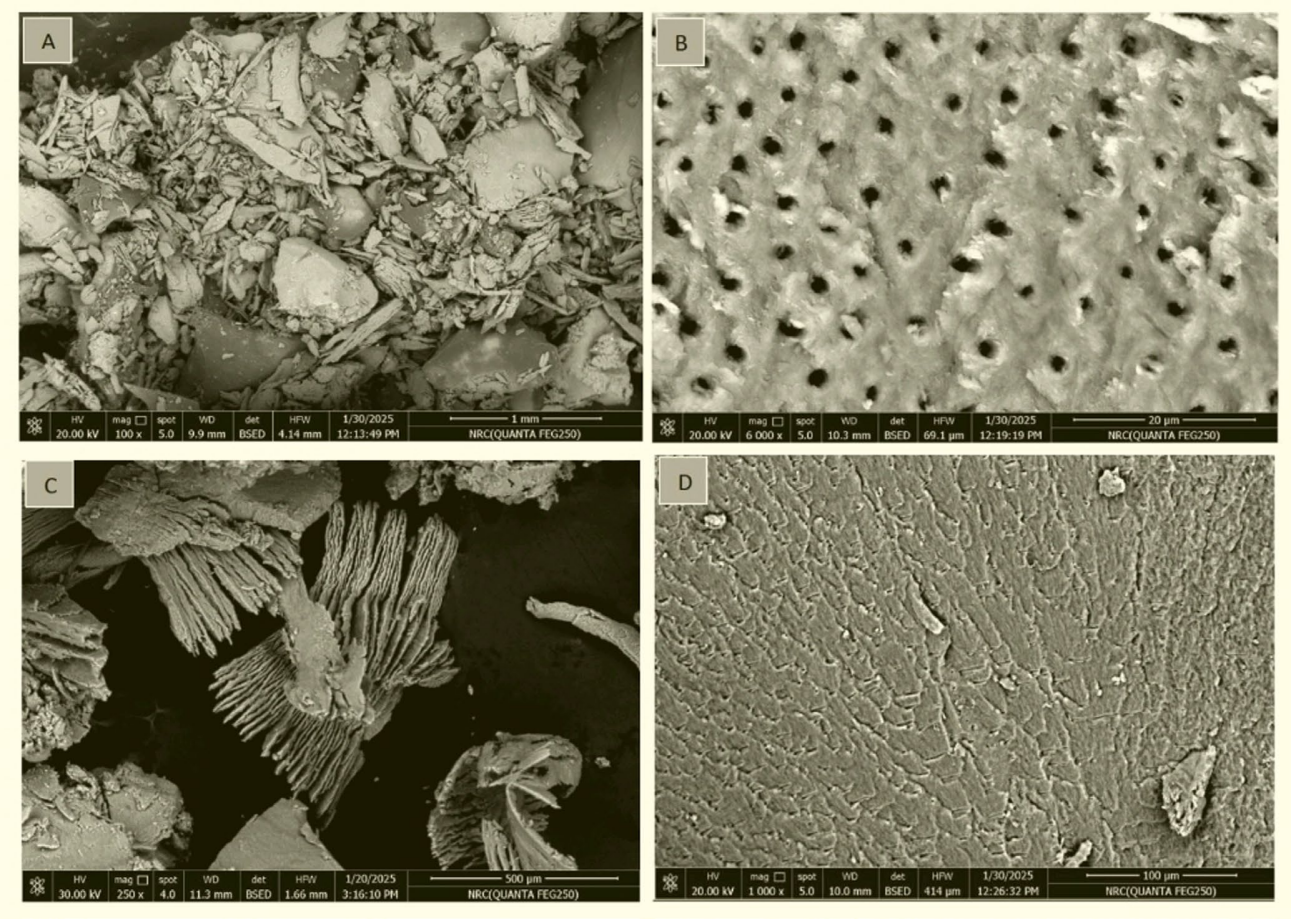
(a) Scanning electron microscopy of demineralized dentin graft particles at 1‐mm magnification; (b) dentinal tubules measurements at 20 μm level; (c) augmented evaluation of collagenized tooth particles at 500 μm level; (d) particle image at 100 μm level.

Following extraction, teeth were cleaned, dried, crushed, ground, and incubated in hydrochloric acid as described above. High‐resolution imaging was performed using a QUANTA FEG250 field‐emission scanning electron microscope (FEI, Thermo Fisher Scientific, Waltham, MA, USA) at the National Research Center, Giza, Egypt. Samples were mounted on SEM stubs with conductive adhesive tape and sputter‐coated with a 10–20 nm layer of gold (Au) or platinum (Pt) using an S150A sputter coater (EDWARDS High Vacuum, Burgess Hill, England) to enhance conductivity and minimize charging without obscuring surface topography. Imaging was conducted at 10–15 kV acceleration voltage, optimized for biological specimens, with an 8–10 mm working distance to balance resolution and depth of field. Morphological assessment was performed at 100× magnification using secondary electron (SE) detection for high‐resolution surface topography. SEM micrographs were subsequently analyzed for surface topography, porosity, and particle homogeneity [36, 37] (Figure 4).

### 2.10 Energy‐Dispersion X‐Ray Spectroscopy (EDS) Analysis

Energy‐Dispersion X‐ray Spectroscopy (EDS) was used to determine the samples' chemical composition. Both spot analysis and area mapping were performed to evaluate mineral distribution for calcium (Ca), phosphorus (P) and oxygen (O). An acceleration voltage of 15 kV was used to optimize X‐ray emission from light elements.

### 2.11 Statistical Analysis

Numerical data were explored for normality by checking the distribution of data and using tests of normality (Kolmogorov–Smirnov and Shapiro–Wilk tests). Radiographic measurement data showed normal (parametric) distribution while changes, percentage changes in radiographic measurements, histomorphometry data, pain and satisfaction scores showed nonnormal (nonparametric) distribution. Data were presented as mean, standard deviation (SD), median and range values. For parametric data, repeated measures ANOVA test was used to compare between the two groups as well as to study the changes within each group. Bonferroni's post hoc test was used for pair‐wise comparisons when the ANOVA test was significant. For nonparametric data, Mann–Whitney *U* test was used to compare between the two groups. The significance level was set at *p* ≤ 0.05. Statistical analysis was performed with IBM SPSS Statistics for Windows, Version 23.0. Armonk, NY: IBM Corp.

The trial was conducted and reported in accordance with the CONSORT guidelines.

## 3 Results

### 3.1 Groups Characteristics

Treatment and follow‐up took place from February 2023 to August 2024. Twenty‐six patients completed their follow‐up, out of the 30 participants, in the current research. The mean age of the 24 female and 6 male patients was 34 ± 6 years. Twelve females and 1 male in the test group, whose mean age was 34 ± 8 years, and 3 males and 10 females in the control group, with a mean age of 35 ± 5 years, completed the study. In terms of the distribution of the extracted teeth, the test group included one mandibular incisor, two mandibular premolars, two maxillary premolars, and eight maxillary incisor teeth, while the control group included nine maxillary incisors, two mandibular incisors, one mandibular premolar, and one maxillary premolar. By the end of the follow‐up period, both groups showed full soft tissue healing and no unanticipated negative effects.

### 3.2 Radiographic Analysis

While the ADDG+HA group exhibited 6.85 ± 1.91 mm BLRW, 9.73 ± 2.91 mm BRH, and 9.2 ± 2.4 mm LRH, the ADDG group demonstrated 6.16 ± 0.66 mm BLRW, 8.74 ± 2.11 mm BRH, and 9.08 ± 2.42 mm LRH at baseline, with no significant differences between the two groups (*p* > 0.05). The ADDG+HA group's mean BLRW loss was 1.18 ± 0.86 mm (17.2% ± 12.5%), while the ADDG group demonstrated a mean BLRW loss of 1.21 ± 0.77 mm (19.6% ± 12.6%; *p* > 0.05) after 6 months. The ADDG+HA group showed a loss of 0.83 ± 0.85 mm (8.6% ± 8.5%) and 1.05 ± 1.15 mm (10.8% ± 11.7%) for BRH and LRH, respectively. The ADDG group incurred a loss of 0.89 ± 0.74 mm (9.2% ± 11.1%) and 0.9 ± 0.76 mm (9.4% ± 8.2%; *p* > 0.05, Table 1).

**TABLE 1:** Baseline measurements and changes in radiographic bone (mm and %).

	ADDG+HA group (test) mean (SD)	ADDG group (control) mean (SD)	*p*
Baseline BLRW (mm)	6.85 (1.91)	6.16 (0.66)	0.250
BLRW loss (mm)	−1.18 (0.86)	−1.21 (0.77)	0.908
BLRW loss (%)	−17.2 (12.5)	−19.6 (12.6)	0.603
Baseline LRH (mm)	9.2 (2.4)	9.08 (2.42)	0.905
LRH loss (mm)	−1.05 (1.18)	−0.9 (0.76)	0.931
LRH loss (%)	−10.8 (11.7)	−9.4 (8.2)	0.083
Baseline BRH (mm)	9.73 (2.91)	8.74 (2.11)	0.353
BRH loss (mm)	−0.83 (0.85)	−0.89 (0.74)	0.298
BRH loss (%)	−8.6 (8.5)	−9.2 (11.1)	0.563

Abbreviations: BLRW, bucco‐lingual ridge width; BRH, buccal ridge height; LRH, lingual ridge height.

### 3.3 Scanning Electron Microscopy (SEM) and Energy Dispersive Spectroscopy (EDS)

SEM and EDS investigations provided insights into the structural and compositional properties of demineralized dentin, detailing its elemental composition: Calcium (Ca), Oxygen (O), and Phosphorus (P) (Table 2). The SEM pictures displayed substantial morphological variations of demineralized dentin. SEM images of the demineralized dentin revealed a smooth and clean surface, with exposed collagen fibrils and open tubules. These findings highlighted the transforming influence of the demineralization process on dentin, making it structurally more comparable to alveolar and cortical bone. Additionally, the particle size examination under SEM at 100× magnification indicated that the demineralized dentin particles varied from 20 to 500 μm, with a porous structure that mimics the osteoconductive architecture of natural bone. This porous shape could be decisive for bone regeneration, since it could enable cell adhesion, penetration, proliferation and vascularization. The exposed collagen networks in demineralized dentin would act as a scaffold for bone tissue development, due to collagen's involvement in promoting cell adhesion, development and differentiation.

**TABLE 2:** Energy dispersive spectroscopy (EDS) results of demineralized dentin graft sample.

Element	Weight %	Atomic %	Net Int.	Error %	Kratio	Z	A	F
Calcium	13.50	20.06	30.36	25.36	0.0369	1.0729	0.2547	1.0000
Oxygen	59.53	66.43	250.01	10.26	0.1272	1.0274	0.2080	1.0000
Phosphorus	11.40	6.57	261.61	3.88	0.0884	0.8977	0.8543	1.0116

*Note:* Weight%: mass fraction of each element, Atomic%: the number of atoms of each element in relation to the total atomic number, Net Int.: intensity of X‐ray signal emitted by the element after accounting for background noise, Error%: an estimate of the accuracy of elemental analysis, Kratio: measurement of relative intensity of element's X‐ray signal, Correction parameters: Z (atomic number correction), A (absorption correction of X‐ray within the sample), F (fluorescence effects).

### 3.4 Histological Evaluation and Histomorphometry Analysis

Microscopic examination of the H&E‐stained sections for the core biopsies in both groups revealed dentin particles embedded in a soft fibro‐vascular connective tissue. Newly formed bone trabeculae with osteocytes in their lacunae were observed surrounding the graft material of variable size. The soft tissue around the bone trabeculae showed abundant osteoblasts. No obvious inflammatory response could be detected surrounding the graft particles. MT‐stained sections of the ADDG+HA group revealed graft particles of variable size embedded in green soft tissue and surrounded by areas of newly formed bone trabeculae. Bone trabeculae were composed of large areas of osseous tissue (mineralized hard bone tissue) of red color, while smaller areas of osteoid tissue (unmineralized soft bone tissue) appeared in green color. The greatest mean value of the mineralized hard bone was detected in the ADDG+HA group which had a mineralized bone area of 33% ± 8.1%, an unmineralized bone area of 12.9% ± 8.3%, a residual graft area of 1.8% ± 0.4% and a soft tissue area of 10.1% ± 4.6%. In contrast, the values for the ADDG group were 14.8% ± 6.5%, 19.1% ± 11.1%, 1.8% ± 0.9% and 15.0% ± 5.6% respectively, with *p* values between the two groups of 0.003, 0.482, 0.110, and 0.338, respectively (Table 3).

**TABLE 3:** Histomorphometry analysis.

	ADDG+HA group (test) mean (SD)	ADDG group (control) mean (SD)	*p*
Mineralized bone (%)	33 (8.1)	14.8 (6.5)	0.003*
Residual graft (%)	1.8 (0.4)	1.8 (0.9)	0.482
Soft tissue (%)	10.1 (4.6)	15 (5.6)	0.110
Un‐mineralized bone (%)	12.9 (8.3)	19.1 (11.1)	0.338

* *p* < 0.05.

### 3.5 Postoperative Pain (VAS Scores) and Patient Satisfaction

There was no statistically significant difference between the two groups regarding VAS pain scores (*p* > 0.05, effect size = 0.201) or overall patient satisfaction (*p* > 0.05, effect size = 0).

## 4 Discussion

Notable alterations in the dimensions of both soft and hard tissues following teeth extraction, could greatly influence prosthetic rehabilitation and treatment planning [38, 39, 40]. ARP approaches aim to minimize bone resorption and thereby preserve the volume and shape of the alveolar ridge following exodontia [41, 42], with a number of approaches being suggested, employing osteogenic or osteoconductive scaffolds, with encouraging outcomes [43, 44]. Yet, results remain inconsistent, with a clear clinical need for exploring new approaches and materials [41, 43]. The present pilot trial aimed to evaluate for the first time the radiographic and histological outcomes of amalgamating HMW‐HA with ADDG for ARP, following tooth extraction in the esthetic zone.

HA's biocompatible, moisturizing, antimicrobial and anti‐inflammatory qualities are believed to positively impact soft tissue repair and bone regeneration [22, 45, 46, 47, 48, 49, 50, 51, 52]. Relying on its beneficial biological, physio‐chemical and osteogenic characteristics [16, 53, 54], following application into the wound, the generated hydrogel‐like matrix would stabilize the blood clot, inhibit soft tissue collapse, speed up epithelialization and collagen deposition, and act as a space maintainer for bone regeneration [20, 55]. HMW‐HA was applied in the treatment of chronically infected extraction sockets [56], and mixed with grafts to increase their stability, hydration and cellular penetration [57] as well as to improve their surgical handling for the treatment of bone defects with challenging defect morphologies [44, 58]. The HMW‐HA gel employed in the current trial was selected based on its reported therapeutic benefits in periodontal applications, demonstrated in a multitude of in vitro, animal and clinical studies. Its formulation with HMW‐HA was shown to promote cell migration, differentiation, angiogenesis and osteoconduction, all critical for tissue regeneration and wound healing [59, 60]. In vitro studies support its low cytotoxicity and positive impact on fibroblast viability [61, 62], while animal models confirmed its role in enhancing osteoblast activity and alveolar bone healing [63]. Clinical trials have further demonstrated its efficacy in reducing inflammation and accelerating healing when used as an adjunct to surgical and nonsurgical periodontal therapy [64, 65, 66] as well as following extraction [67]. Although the employed 0.2% Gengigel is a commercially available product and its proprietary formulation includes excipients beyond HA, which could (even if minutely) contribute to the observed biological effects, its consistent performance across multiple study types demonstrates its clinical relevance.

The addition of cross‐linked HA to grafting materials during bone regeneration approaches was shown to increase the amount and quality of resultant bone [68, 69] with even dispersion, greater mineralization density of the freshly formed bone [44, 57, 70, 71]. Yet contrastingly, a recent systematic review showed that HA might not offer additional advantages regarding new bone formation and remaining graft particles when paired with grafting materials histomorphometrically [72]. Variations in research design, HA characteristics, outcome measures, graft material and patient characteristics, are generally believed to affect HA's activity [73].

In the current trial, in the test group a combination of HMW‐HA with ADDG did not exert a significant effect on bone volume of the alveolar ridge compared to the control group, with both groups experiencing similar alterations in buccal and palatal wall heights and bucco‐lingual socket width. Similar to previous investigations demonstrating the beneficial effects of HA on postoperative inflammation and related symptoms [74], both groups reported comparable pain scores with similar satisfaction levels. SEM examination of the ADDG at the micro‐ and nanoscale level demonstrated that demineralized dentin had a porous, collagen‐rich structure resembling natural bone, whereas EDS analysis proved its chemical resemblance to demineralized bone. The presence of calcium and phosphorus suggests that the ADDG retains its bioactive properties useful for osteoconductive functions in addition to its harbored osteoinductive dentin‐specific growth factors. Consistent with earlier findings on HA's osteoinductive, osteoconductive qualities and its ability to promote osteoblastic activity and mineralization processes [44, 75], histologically in the present trial HA revealed an augmenting effect on bone maturation, with improved quality of the newly formed bone in the sockets receiving HMW‐HA mixed with the growth factors‐laden demineralized ADDG. Similar to earlier findings on combining HA with demineralized freeze‐dried bone allograft (DFDBA) [76], the ADDG+HA group displayed significantly greater areas of mineralized bone, suggesting that HA fosters bone mineralization/maturation when combined with demineralized bone grafts, a process essential for the structural integrity and mechanical strength of newly formed bone [77]. In contrast to the ADDG group, the present findings similarly suggest that HMW‐HA contributed to greater areas of mineralized bone, by potentially providing abundant nucleation sites for mineral deposition and creating an optimal microenvironment for osteoblast activity [78]. In line with previous reports [79] it is thus plausible to carefully assume, that the presence of HMW‐HA in conjunction with ADDG, might positively influence the quality of the newly regenerated bone during ARP, with possibly better long‐term perspective of the subsequently placed implants.

Yet, the present results should be carefully interpreted in light of the current trial's limitations. Firstly, ADDG can only be sourced from individuals who require tooth extractions, which limits its obtainability. This restriction is similar to that observed with autogenous bone grafts, where the quantity of obtainable material is inherently constrained by the tissue availability from the patient. Secondly, ADDG is not a viable grafting option for patients with edentulous regions. This limitation effectively excludes a significant portion of the patient population who could otherwise benefit from ADDG grafting procedures in the course of dental implants or other periodontal/surgical treatments. Thirdly, the current study's focus on single‐rooted teeth and certain socket morphology further limits the generalizability of the results. Fourthly, another challenge associated with dentin grafts lies in the technical sensitivity of its demineralization process. The process, which is crucial for preparing the material for clinical use, might resorb particles unevenly, depending on their size and composition. Fifthly, the reliance on chair‐side bone mills in the absence of sieving, as was the case in the current trial, could result in a variation in the resultant particle size. Such inconsistencies, although these particles were completely demineralized prior to insertion in the defects, could compromise the predictability of the grafting procedure, underscoring the need for highly standardized and precise processing protocols. Additionally, a qualitative analysis of the formed tissues relying solely on a number of histological analyses and in the absence of long‐term follow‐up following the dental implants insertion and loading may not capture the full complexity of bone healing. Furthermore, the use of a commercially available product may additionally introduce limitations in terms of experimental reproducibility and comparison with other HA formulations. Although the tested HMW‐HA formulation has shown promising results in preclinical in vitro studies [62], animal models [63], and clinical trials [60, 65] the proprietary nature of the product introduces uncertainty regarding the attribution of observed effects solely to HA. Therefore, although the employed commercially available HMW‐HA offers practical advantages in terms of safety and ease of use, the observed outcomes of this study should be interpreted with caution, as unexplored effects of the constituents and stabilizers beyond the HMW‐HA in the formulation, could have possibly interacted with the dentin graft material and could have contributed to the observed clinical effects. Furthermore, the molecular weight distribution of HA in the commercially available formulation remains not fully specified, making replication and comparison with other HA formulations difficult. Future research should thus consider using research‐grade HA formulations with fully characterized molecular profiles to enhance scientific rigor and reproducibility. Finally, the high dropout rate in the current trial could have affected the study's power and masked possible clinical effects of the HMW‐HA with ADDG amalgamation.

These constraints collectively highlight the critical need for further research and innovation in the field of employment of dentin grafts, where an optimization of processing methods is paramount to ensure consistent material quality and predictable clinical outcomes. Furthermore, exploring alternative sources for bone grafting in oral and maxillofacial procedures remains a vital area of investigation to overcome the inherent limitations of autogenous materials and expand the accessibility of effective grafting solutions.

## 5 Conclusions

Given the current pilot trial's limitations, it can be concluded that ADDG mixed with HMW‐HA enhances bone healing potential, with better bone quality for subsequent implant placement, yet without a positive effect on dimensional changes during ARP procedures. By enhancing both the quantity and quality of mineralized bone, HA appears to augment the osteoconductive and chemotactic properties of ADDG, leading to a more robust and mature bone repair. Further, it can be plausibly inferred that HMW‐HA could boost the clinical outcomes in bone grafting procedures. Further randomized controlled studies with longer follow‐up periods, larger sample sizes and different graft materials, as well as ARP trials on the effect of HA alone, in compromised and multirooted teeth are recommended.

## Author Contributions

R.H. conceived the idea, performed the surgeries and wrote the initial draft of the manuscript. N.S. supervised the surgical procedures, collected and analyzed the data, and critically reviewed the manuscript. K.F.E.‐S. supervised the surgical procedures, critically analyzed the results and led the writing of the manuscript. H.A.S. performed the histomorphometry analysis and reviewed the manuscript. All authors reviewed the manuscript, gave their final approval and agreed to be accountable for all aspects of the work.

## Conflicts of Interest

The authors declare no conflicts of interest.

## Supporting information


**Figure S1:** Patients' recruitment.

## Data Availability

All data of the manuscript is available from the corresponding author upon reasonable request.
