# Supplementary material for: Autogenous Demineralized Dentin Graft With High Molecular Weight Hyaluronic Acid in Ridge Preservation: Pilot Trial
Source: Clin Implant Dent Relat Res. 2025 Nov 14;27(6):e70100. doi: 10.1111/cid.70100 (PMC12616775; doi:10.1111/cid.70100)
Supplement: Supplementary file 1 — Figure S1: Patients' recruitment. [file CID-27-0-s001.docx]

**Figure S1:** Patients’ recruitment

**CONSORT 2010 Flow Diagram**

Lost to follow-up (refused to attend) (n=2)

Discontinued intervention (give reasons) (n=0)

## **Follow-Up**

Analysed (n=13)
♦ Excluded from analysis (give reasons) (n=0)

## **Analysis**

Analysed (n=13)
♦ Excluded from analysis (give reasons) (n=0)

Lost to follow-up (refused to attend) (n=2)

Discontinued intervention (give reasons) (n=0)

## **Enrollment**

Allocated to test group (n=15)

♦ Received allocated intervention (n=15 )

♦ Did not receive allocated intervention (give reasons) (n=0)

## **Allocation**

Allocated to control group(n=15)

♦ Received allocated intervention (n=15)

♦ Did not receive allocated intervention (give reasons) (n=0 )

Randomized (n=30)

Excluded (n=40)

♦  Not meeting inclusion criteria (n=34)

♦  Declined to participate (n=6)

Assessed for eligibility (n=70)
